# Supplementary material for: Evaluation of Thirdhand Smoke Exposure after Short Visits to Public Facilities (Noraebang and Internet Cafés): A Prospective Cohort Study
Source: Toxics. 2022 Jun 7;10(6):307. doi: 10.3390/toxics10060307 (PMC9227155; doi:10.3390/toxics10060307)
Supplement: Supplementary file 1 [file toxics-10-00307-s001.zip › toxics-1699458-supplementary.pdf]

toxics

Article

Evaluation of Thirdhand Smoke Exposure after Short Visits to Public Facilities (Noraebang and Internet Cafés): A Prospective Cohort Study

Myung-Bae Park [1] and Boram Sim [2,*]

[1] Department of Gerontology Health and Welfare, Pai Chai University, Daejeon 35345, Korea; parkmb@pcu.ac.kr
[2] HIRA Research Institute, Health Insurance Review and Assessment Service (HIRA), Wonju 26465, Korea
* Correspondence: simbr12@hira.or.kr

Supplementary Files

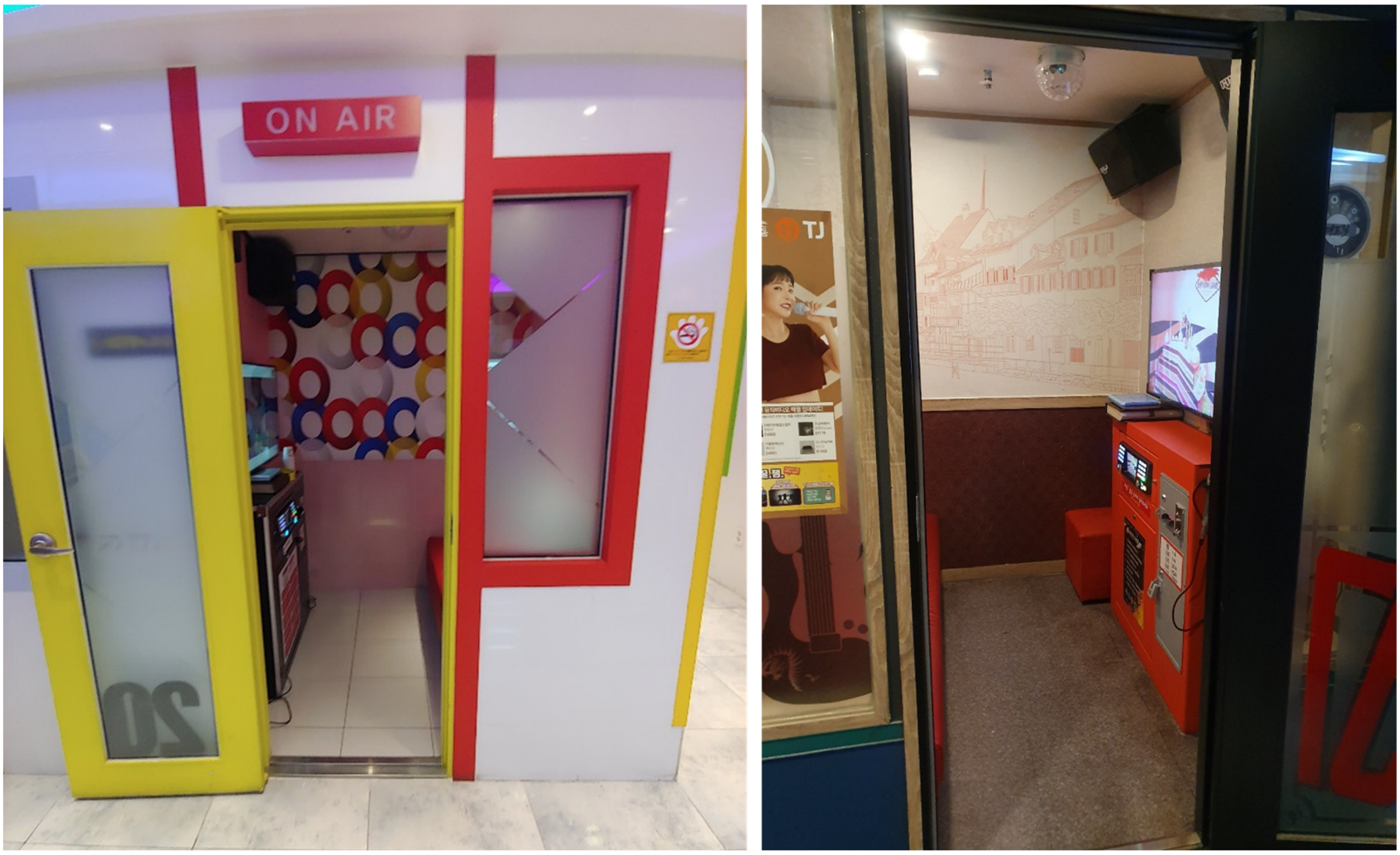

Figure S1. Target Venue (Noraebang, Korean style karaoke)

* In noraebangs are not currently legal non-smoking areas. And use the facility by one person or one team in a completely separate space.

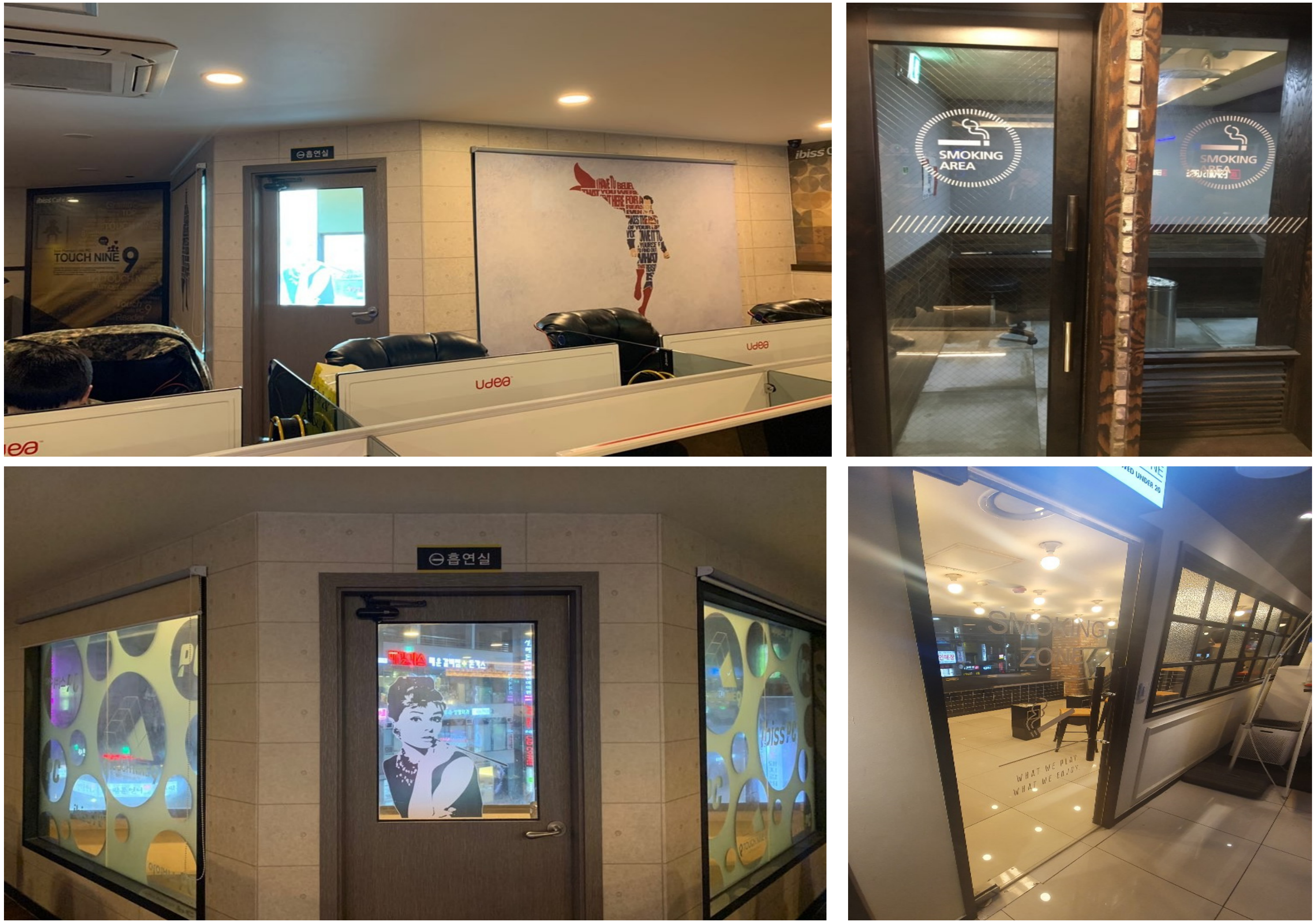

Figure S2-1 Smoking area of Target Venue (PC café): Outside view of indoor smoking area**.**

* In Korea, PC café has been designated as an utterly non-smoking area since 2014, and a separate room can be designated as a smoking area. However, a smoking area can be installed, and according to the law, the space must be completely separated, and a ventilation system must be installed and operated at all times to allow air to escape to the outside in the smoking room. It is similar to the indoor smoking area of an airport.

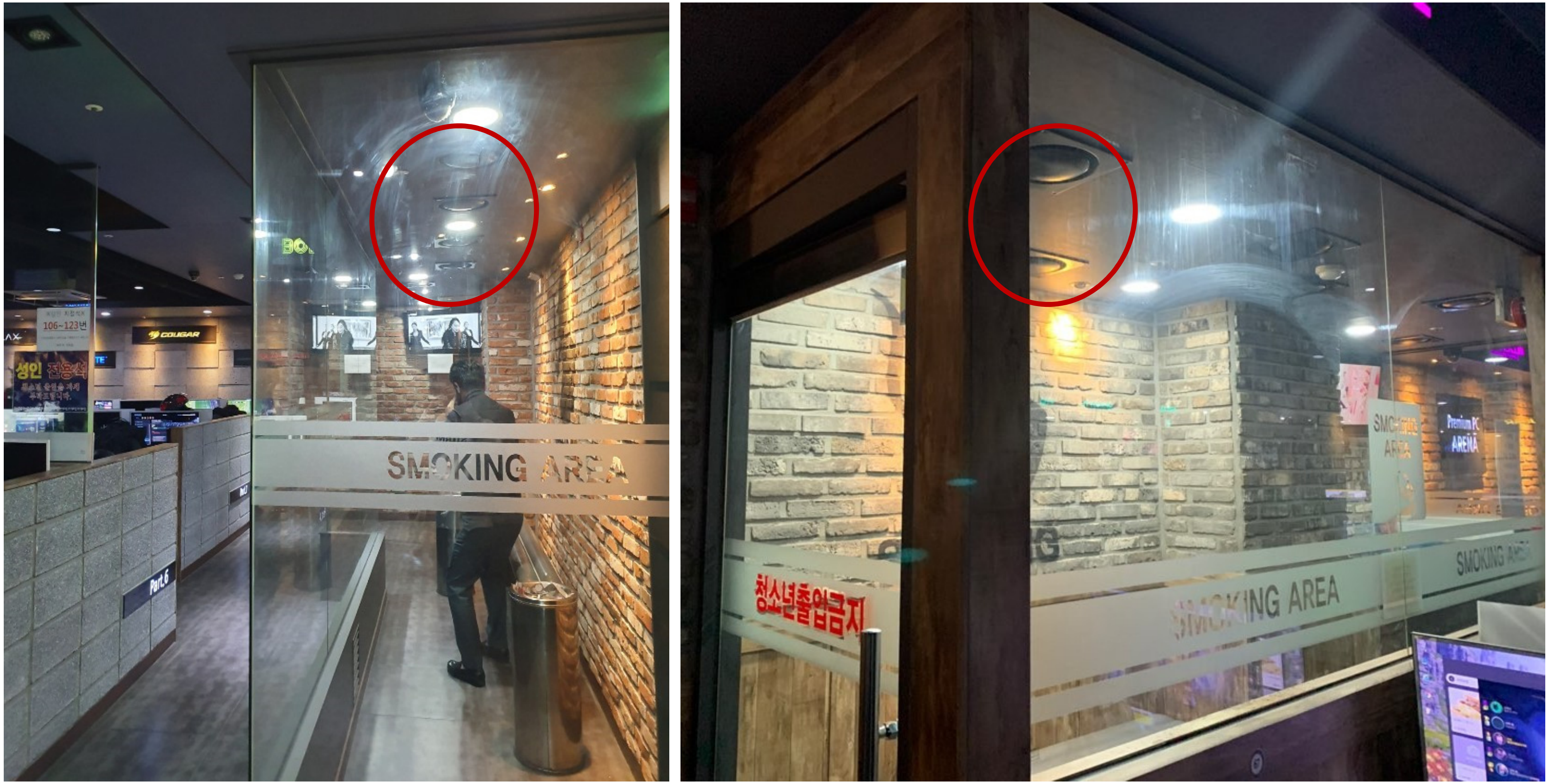

Figure S2-2. Smoking area of Target Venue (PC café): Ventilation system.

* The red circle is the ventilation system that draws air to the outside.

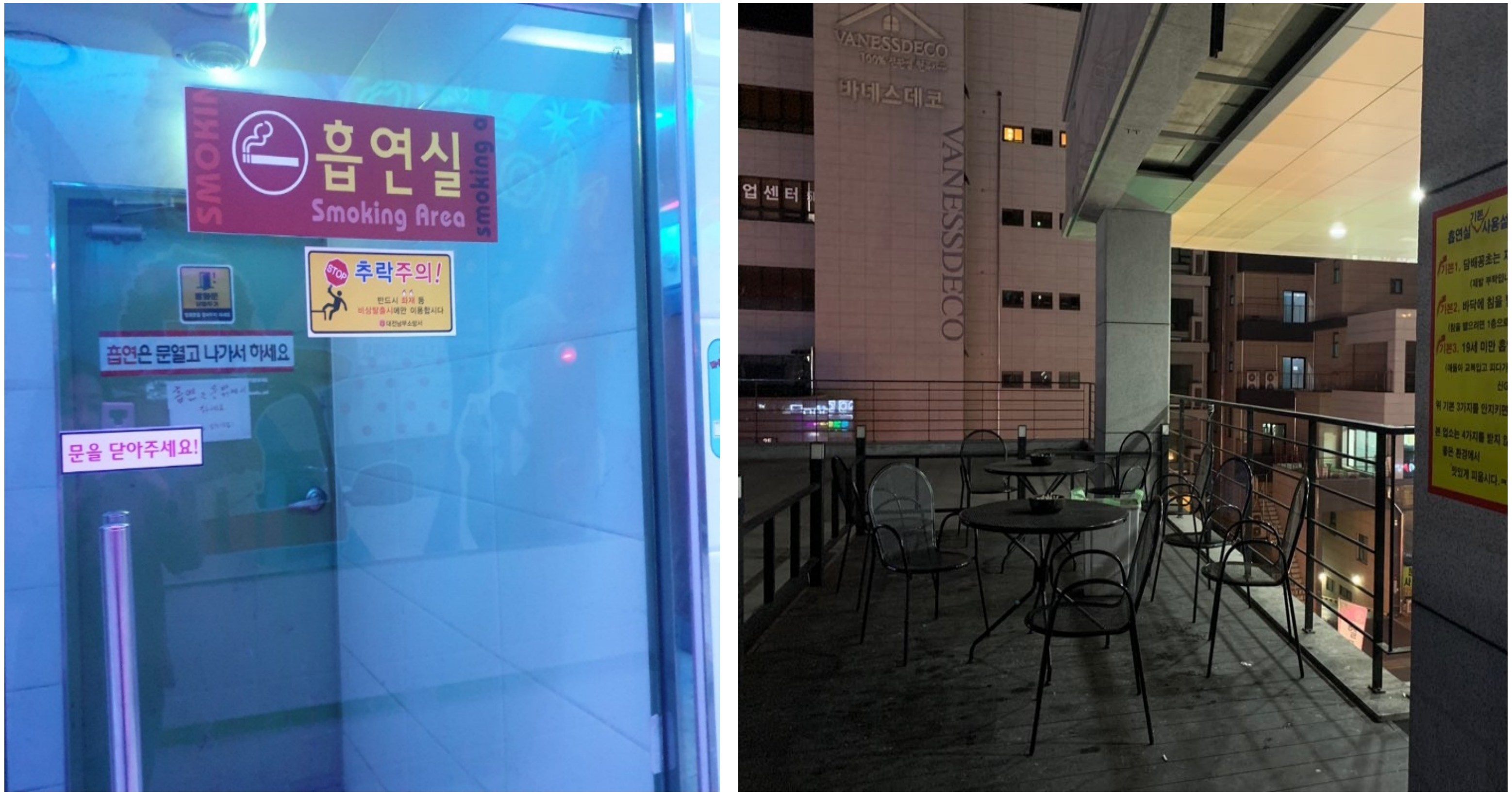

Figure S2-3. Smoking area of Target Venue (PC café): Outdoor smoking area connected to the facility

* In places with a terrace or balcony, a smoking area may be installed outside, as shown in the photo. The door in the photo on the left connects to the outdoor smoking area on the right.
